# Association of *MAP2* gene polymorphisms and altered expression with schizophrenia risk in a Chinese Han population

**DOI:** 10.3389/fpsyt.2026.1844302

**Published:** 2026-06-29

**Authors:** Mengyi Yang, Jia Yu, Yucan Chang, Yurui Chen, Wenmei Xie, Liqiong Liu, Zhenghao Huo, Zhanbing Ma, Jie Dang

**Affiliations:** 1 School of Basic Medicine, Ningxia Medical University, Yinchuan, Ningxia, China; 2 School of Basic Medicine, Gansu Medical College, Pingliang, Gansu, China; 3 Key Laboratory of Fertility Preservation and Maintenance of the Ministry of Education, Ningxia Medical University, Yinchuan, Ningxia, China

**Keywords:** gene expression, MAP2, negative symptoms, schizophrenia, SNP

## Abstract

**Background:**

Schizophrenia (SCZ) is a highly heritable primary psychotic disorder. The microtubule-associated protein 2 (*MAP2*) gene is essential for dendritic integrity and synaptic plasticity, positioning it as a key candidate for bridging genetic risk and neuropathology. Nevertheless, the role of common genetic variations within *MAP2* in SCZ susceptibility remains to be elucidated.

**Methods:**

We conducted a candidate gene association study of *MAP2* in a Han Chinese cohort comprising 418 SCZ patients and 418 matched healthy controls. Targeted sequencing was used to genotype single nucleotide polymorphisms (SNPs). *MAP2* mRNA levels were quantified by RT-qPCR and correlated with genotypes and clinical symptoms. Bioinformatic tools (such as GTEx, BrainSeq, 3DSNP, HaploReg, RegulomeDB and SNP2TFBS database) were employed for functional annotation of risk loci.

**Results:**

We identified multiple *MAP2* SNPs associated with SCZ risk in a Han Chinese cohort. Specifically, the AA genotype of rs288057 and the GG genotype of rs288087 were significantly associated with increased disease risk (OR = 2.393 and 2.258, respectively). Expression analysis revealed a marked reduction in peripheral *MAP2* mRNA levels in patients compared to controls. This downregulation was genotype-dependent: the risk AA at rs288057 and GG at rs288087 were correlated with lower mRNA levels, a finding supported by its significant eQTL effect in the GTEx and BrainSeq database. In silico annotation suggested rs288087 resides within a putative enhancer region, while rs288057 may affect a promoter-proximal regulatory site. Clinically, *MAP2* expression showed a significant positive correlation with the severity of negative symptoms (SANS score). Furthermore, ROC analysis indicated that *MAP2* expression levels distinguished patients from controls with an AUC of 0.728.

**Conclusion:**

This study identifies *MAP2* as a schizophrenia risk gene, wherein non-coding variants likely reduce its expression via distinct regulatory mechanisms, linking this downregulation to core negative symptoms. These findings highlight *MAP2*’s pathophysiological and translational relevance.

## 1 Introduction

Schizophrenia (SCZ), a severe psychiatric condition, affects approximately 1% of the global population, exerting significant personal, familial, and societal burdens (1). The origins of SCZ are closely linked to genetic factors, with heritability rates ranging from 60% to 85% depending on the genetic relationship and the number of affected family members (2). Extensive genomic research, especially genome-wide association studies (GWAS), has uncovered numerous common risk loci, confirming SCZ as a polygenic illness and highlighting biological pathways essential for synaptic activity, neuronal communication, and brain development (3–7). However, a limitation of relying solely on stringent GWAS significance thresholds is the potential to overlook biologically relevant genes with modest effect sizes or population-specific effects. This is particularly pertinent for genes regulating fundamental neuronal architecture, such as microtubule-associated protein 2 (*MAP2*). Although large-scale GWAS have not identified *MAP2* as a genome-wide significant locus, its critical and well-established role in dendritic architecture and neuronal structural integrity provides a compelling biological rationale for hypothesis-driven investigation. Importantly, *MAP2* function can be altered by epigenetic and post-translational modifications (e.g., phosphorylation) that escape GWAS detection (8, 9). Hypothesis-driven investigations of these candidates are therefore critical for bridging the gap between genetic association and disease mechanisms.

A leading model for understanding SCZ is the neurodevelopmental hypothesis. This theory suggests that the interplay between genetic susceptibility and environmental stressors disrupts typical brain development, resulting in a vulnerability that manifests as psychosis in early adulthood (10, 11). The studies have identified that the aberrant maturation of neural circuits, particularly in the cortical circuits, is a key factor contributing to the characteristic dysconnectivity observed in SCZ (12, 13). At the cellular scale, the precise coordination of neurodevelopmental events, such as neuronal proliferation, migration, polarization, dendritic arborization, and synaptogenesis, is critically dependent on the dynamic reorganization of the cytoskeleton, particularly the microtubule framework. This is evidenced by the role of microtubule polarity in guiding cell migration and the importance of microtubule dynamics in neuronal migration (14, 15).

Microtubules, as integral components of the neuronal cytoskeleton, play a crucial role in maintaining neuronal structure and function. They serve as essential scaffolds and primary pathways for the transport of cellular materials, which is vital for establishing and sustaining neuronal polarity, morphology, and synaptic connections (16). This regulation is critical for microtubules to maintain cellular structure, facilitate transport, and enable the complex morphological changes underlying neuronal development and function (17). *MAP2* is a neuron-specific microtubule binding protein, is predominantly localized to the somatodendritic compartment, as evidenced by its critical role in maintaining neuronal structure and function (18). It plays an indispensable role in promoting microtubule assembly, stabilization, and bundling, which are fundamental processes for the elaboration and maintenance of complex dendritic arbors (8). As dendrites constitute the principal site of synaptic input and integration, *MAP2* is essential for shaping the neuronal receptive field and computational capabilities (19). Consequently, *MAP2* is widely regarded as a canonical biomarker of neuronal differentiation and dendritic integrity, with alterations in its expression, localization, or post-translational modifications implicated in the pathophysiology of various neurodevelopmental and psychiatric disorders (20–22).

Converging evidence from post-mortem studies consistently reveals dendritic abnormalities, such as reduced spine density and simplified arborization, in key brain regions like the prefrontal and hippocampal cortices in SCZ (23, 24). A meta-analysis of 31 post-mortem studies confirmed a significant reduction of dendritic spine density in the prefrontal cortex of SCZ patients (25), and recent updates to the synaptic hypothesis continue to support these findings (7). These structural deficits are frequently accompanied by significant reductions in *MAP2* protein and mRNA levels (26, 27). This strong correlation positions *MAP2* deficiency as a pivotal molecular link, potentially connecting upstream neurodevelopmental insults to the downstream synaptic and circuit dysconnectivity that characterizes SCZ. However, a critical question remains. Do common genetic variants within *MAP2* contribute to disease susceptibility by directly regulating its expression? Elucidating this genetic mechanism is essential to move beyond correlation and establish a causal understanding of the disorder’s etiology.

Here, we perform a comprehensive genetic and functional analysis of *MAP2* in a Chinese Han population with SCZ. We hypothesize that functional polymorphisms at this locus influence disease susceptibility by modulating its expression. To test this, we integrate single nucleotide polymorphisms (SNPs) based association, haplotype analysis, and genotype-specific expression assessment. This work directly bridges genetic variation at the *MAP2* locus with its transcriptional regulation and disease risk, providing critical mechanistic evidence that links a key cytoskeletal regulator to the genetic and neurodevelopmental basis of SCZ, and identifies a new potential target for therapeutic intervention.

## 2 Methods

### 2.1 Study participants

A cohort of 418 patients diagnosed with SCZ (202 males, 216 females; mean age 46.24 ± 12.56 years) was enrolled at Ning’an Hospital and Minkang Hospital in Ningxia, China between 2024 and 2025. Comprehensive demographic and clinical data were collected. All patients fulfilled the diagnostic criteria for SCZ according to both the Diagnostic and Statistical Manual of Mental Disorders, Fourth Edition (DSM-IV) and the Chinese Classification and Diagnostic Criteria of Mental Disorders, Third Edition (CCMD-3). Exclusion criteria included severe systemic illness, a history of abuse or dependence on alcohol, tobacco, or other psychoactive substances, secondary psychotic disorders, organic brain pathologies, and any condition resulting in communication barriers, cognitive impairment, or non-compliance that could compromise successful sample procurement. A systematic collection of general demographic and clinical data was conducted for all participants. General data, including age, ethnicity, gender, place of residence, occupation, family history of genetic diseases, and handedness, were collected using a structured demographic questionnaire. Clinical data were obtained through the administration of six standardized symptom rating scales: the Brief Psychiatric Rating Scale (BPRS), the Scale for the Assessment of Positive Symptoms (SAPS), the Scale for the Assessment of Negative Symptoms (SANS), the Young Mania Rating Scale (YMRS), the Hamilton Depression Rating Scale (HAMD), and the Hamilton Anxiety Rating Scale (HAMA).

A control group of 418 healthy individuals (188 males, 230 females; mean age 44.90 ± 14.00 years) was concurrently recruited from individuals undergoing routine health assessments at the General Hospital of Ningxia Medical University. All control participants were screened to confirm the absence of any personal history of psychiatric disorders, diabetes, hypertension, endocrine diseases, malignancies, or neurological conditions, and no history of dependence on alcohol, tobacco, or other drugs. All study subjects were confirmed to be biologically unrelated.

The study protocol received approval from the Ethics Committee of Ningxia Medical University (Approval No.: 2022-G185). Written informed consent was obtained from every participant following a thorough explanation of the study’s objectives and procedures. For subsequent genetic analysis, all patients provided fasting peripheral venous blood samples on the first day of hospital admission, prior to the initiation of any antipsychotic medication. Healthy control volunteers, recruited during the same period, had not taken any psychotropic medications before blood collection. Approximately 2 mL of peripheral venous blood was drawn from each participant into EDTA-anticoagulated vacuum tubes.

### 2.2 SNP selection

Potential SNPs within the *MAP2* gene locus were identified using the SNPinfo Web Server (http://snpinfo.niehs.nih.gov/). The selection criteria were as follows: 1) the SNP must be a tagSNP, located within 1000 kb upstream or downstream of the gene, including exons, introns, 5’-UTR, and 3’-UTR regions; 2) the minor allele frequency (MAF) must be at least 0.05 in the East Asian population; and 3) priority was given to SNPs with previously reported significant associations in related literature. Based on these criteria, 8 SNPs (rs13017199, rs561828230, rs997109, rs16843019, rs2365661, rs288057, rs288087 and rs741006) were selected for further investigation. Detailed information on these SNPs is provided in **Table 1**.

**Table 1:** Basic information on the 8 SNP loci of the *MAP2*.

No.	SNP	Chromosome	Position	Allele	Region	1000G_CHB
1	rs13017199	2	210246064	C/G	intergenic	0.23
2	rs561828230	2	210288886	C/T	UTR5	0.17
3	rs997109	2	210341429	C/T	intronic	0.49
4	rs16843019	2	210382435	C/T	intronic	0.25
5	rs2365661	2	210391837	A/G	intronic	0.10
6	rs288057	2	210392711	A/G	intronic	0.74
7	rs288087	2	210421459	A/G	intronic	0.40
8	rs741006	2	210558162	G/A	exonic	0.20

### 2.3 DNA extraction

Genomic DNA was extracted from whole-blood samples using a commercial Blood Genomic DNA Extraction Kit (TIANGEN Biotech, Beijing, China) following the manufacturer’s protocol. DNA concentration and purity were assessed using a full-wavelength microplate reader (Thermo Fisher Scientific, Vantaa, Finland). Samples with an A260/A280 ratio between 1.8 and 2.0 were considered of high quality and stored at –20 °C for subsequent genotyping.

### 2.4 Genotyping

Eight selected SNPs within the *MAP2* gene were genotyped using target capture sequencing (Shanghai Tianhao Biotechnology Co., Ltd., China). To ensure genotyping accuracy, approximately 5% of samples (n=40) were randomly selected for repeat genotyping. The concordance rate between initial and repeated results was 100%, confirming high reproducibility and reliability of the genotyping data.

### 2.5 Reverse transcription quantitative PCR (RT-qPCR) analysis

Peripheral blood mononuclear cells (PBMCs) were isolated from a randomly selected subset of participants. Total RNA was extracted using TRIzol reagent (Thermo Fisher Scientific, USA) and reverse-transcribed with the PrimeScript RT Reagent Kit (TaKaRa, Dalian, China). RT-qPCR was performed on a 7500 RT-qPCR System (Applied Biosystems, USA) using SYBR Green master mix (Thermo Fisher Scientific, USA). The cycling protocol consisted of an initial denaturation at 95 °C for 30 s, followed by 40 cycles of 95 °C for 15 s and 60 °C for 30 s. To investigate the expression effects of *MAP2* and its significantly associated SNPs, this study was conducted at three levels using subsets of the total SCZ cohort (N = 418) based on genetic and clinical data availability: 1) gene-specific expression analysis was performed in 69 patients randomly selected from the 418 SCZ patients, with the requirement that all three MAP2 genotypes (major homozygote, heterozygote, minor homozygote) were represented to enable statistical comparison. To minimize potential confounding, all 69 selected patients were never-smokers and never-drinkers; 2) *MAP2* expression levels were compared between 31 healthy controls and 31 SCZ patients (the latter randomly selected from the 69 genotyped patients) who had high-quality RNA and were matched for age and sex; and 3) the correlation between *MAP2* mRNA expression levels and clinical symptoms was analyzed in 29 patients from the genotyped subset who had complete clinical symptom rating scales (the remaining 40 patients lacked complete clinical data). *MAP2* expression levels were normalized to the endogenous control GAPDH and calculated using the 2^–Δ*CT* or 2^–ΔΔ*CT* method.

The primers for RT-qPCR were designed using the NCBI Primer-BLAST software (https://www.ncbi.nlm.nih.gov/tools/primer-blast/) and synthesized by Sangon Biotech Co., Ltd. (Shanghai, China). The primer sequences used were:

*MAP2* forward: 5’-CTTCACGCACACCAGGCA-3’.
*MAP2* reverse: 5’-CTCGGCACCAAGATGGCAGAC-3’.
*GAPDH* forward: 5’-CAGGAGGCATTGCTGATGAT-3’.
*GAPDH* reverse: 5’-GAAGGCTGGGGCTCATTT-3’.

### 2.6 Bioinformatics analysis

After a series of statistical analyses, only two SNPs, rs288087 and rs288057 were found to be significantly associated with schizophrenia. To elucidate the expression profile and regulatory mechanisms of *MAP2* and its associated risk SNPs (rs288087 and rs288057) in SCZ, we queried a comprehensive series of bioinformatic analyses using publicly available databases and tools.

#### 2.6.1 Interrogation of public transcriptomic databases (GTEx and BrainSeq) for *MAP2* expression

We retrieved the expression pattern of *MAP2* across 54 human tissues using data from the Genotype Tissue Expression (GTEx) database (version 8.0; https://www.gtexportal.org/home/). To compare its brain expression, dorsolateral prefrontal cortex (DLPFC) transcriptomic data from the BrainSeq database (http://eqtl.brainseq.org/) were used to extract precomputed differential expression results between schizophrenia patients and healthy controls.

#### 2.6.2 Retrieval of eQTL and chromatin interaction data for rs288087 and rs288057 with *MAP2* from public databases

Expression quantitative trait locus (eQTL) associations were retrieved to assess the correlation between the target SNPs and *MAP2* expression: data for rs288057 were obtained from BrainSeq RNA-seq data, while for rs288087 (unavailable in BrainSeq), the GTEx database was queried to extract its tissue-wide association with *MAP2* expression. The 3DSNP portal (version 2.0; https://3dsnp.omic.tech/) was used to retrieve potential chromatin loop interactions involving the SNP loci.

#### 2.6.3 Retrieval of regulatory element and transcription factor binding site annotations for rs288087 and rs288057 from public databases

To evaluate the potential regulatory function of the target SNP, preliminary annotation of genomic features was performed using the HaploReg (version 4.2; https://pubs.broadinstitute.org/mammals/haploreg/haploreg.php) and SNP2TFBS (http://ccg.vital-it.ch/snp2tfbs/) database. Bioinformatic analysis was then conducted using the RegulomeDB database (version 2.2; https://www.regulomeDB.org/), which integrates large-scale chromatin immunoprecipitation sequencing (ChIP-seq) data and chromatin state annotations from projects such as ENCODE. The specific analysis included (1): retrieving ChIP-seq data for the SNP locus to identify transcription factors (e.g., CTCF) potentially binding at this site; and (2) querying chromatin states in various cell types and tissues based on the Core 15-state model (e.g., promoters, enhancers, heterochromatin, quiescent regions) to determine whether the SNP resides within potential regulatory elements.

### 2.7 Statistical analysis

Propensity score matching (PSM) (1:1 nearest neighbor) was performed using the MatchIt package in RStudio (version 4.4.3) to balance age and sex between groups. Genetic association analyses were conducted as follows: Hardy-Weinberg equilibrium (HWE) was assessed in controls using Haploview (version 4.2); case-control association analysis was performed using PLINK (version 1.9); linkage disequilibrium and haplotype analysis were conducted on the SHEsis platform (http://analysis.bio-x.cn) (strong LD defined as D’>0.7 and r^2^>0.5). Gene expression analyses were performed using SPSS (version 25.0; IBM Corp.). Normality was assessed using the Shapiro-Wilk test, and homogeneity of variances was assessed using Levene’s test (significance level α=0.05). All continuous variables met the assumptions of normality (all Shapiro-Wilk *P*>0.05) and homoscedasticity (all Levene’s *P*>0.05). Accordingly, differences between two groups were compared using independent samples t-test; differences among multiple groups were assessed by one-way ANOVA followed by LSD *post hoc* test; correlations were analyzed using Pearson’s correlation coefficient and multiple linear regression analysis; the diagnostic value of *MAP2* expression for schizophrenia was evaluated by receiver operating characteristic curve analysis. Graphs were generated using GraphPad Prism (version 8.0). A two-tailed *P* < 0.05 was considered statistically significant. To control for type I error due to multiple comparisons, For the primary confirmatory analyses (i.e., single SNP association tests including genotype, allele, and genotypic models for the 8 candidate SNPs), Bonferroni correction (28) was applied with k=8, defining statistical significance as *P* < 0.05/8 = 0.006. For secondary exploratory analyses, including haplotype analyses, genotype-specific expression, ROC curve evaluation, and symptom correlation analyses, nominal *P* values are reported without correction for multiple comparisons. These exploratory findings are interpreted with caution and are considered hypothesis-generating, requiring independent replication in future studies.

## 3 Results

### 3.1 Comparison of baseline characteristics between the case and control groups before and after PSM

A total of 1,696 participants were enrolled in this study, including 425 SCZ patients in the case group and 1,271 controls in the healthy group. Before PSM, there was a statistically significant difference in age between the two groups (*P=*5.52e-91), while no significant difference was observed in sex distribution (*P* = 0.482). After PSM, neither age nor sex showed statistically significant differences between the two groups (age: *P* = 0.097; sex: *P* = 0.332). Consequently, a total of 836 participants were included in the final analysis, comprising the case group (males: 202; females: 216) and the healthy control group (males: 188; females: 230), as shown in **Table 2**.

**Table 2:** Comparison of general characteristics before and after PSM.

Variable	Group	Before matching				After matching			
		SCZ	HC	*χ^2^*	*P*	SCZ	HC	*χ^2^*	*P*
N		425	1271			418	418		
Gender	Male	209	600	0.495	0.482	202	188	0.942	0.332
	Female	216	671			216	230		
Age	<30	49	853	426.389	5.52e-91^***^	48	70	7.859	0.097
	30-	82	89			79	86		
	40-	98	96			97	95		
	50-	140	114			138	109		
	60-	56	119			56	58		
Smoking	Yes	77	62	74.200	7.06e-18^***^	75	43	10.104	0.001^**^
	No	348	1209			343	375		
alcohol consumption	Yes	42	22	58.281	2.27e-14^***^	41	17	10.671	0.001^**^
	No	383	1249			377	401		
handedness	Left-handed	31	31	33.452	5.44e-8^***^	29	21	4.900	0.086
	Mixed-handed	18	19			17	8		
	Right-handed	376	1221			372	389		

^**^ *P*<0.01.

^***^ *P*<0.001.

SCZ, schizophrenia; HC, healthy control.

### 3.2 Genotype and allele frequency distribution of 8 SNPs of *MAP2*

Potential SNPs within the *MAP2* gene locus were screened. The genotype distributions of all eight investigated *MAP2* SNPs were in HWE in the control group (*P*>0.05), indicating no significant population stratification. As summarized in **Table 3**, genotype analysis revealed significant differences in frequencies between SCZs and healthy controls at two loci: rs288057 (*P* = 0.008) and rs288087 (*P* = 0.010). After adjusting for confounding factors (sex, age, smoking, alcohol use, and handedness), the significance persisted for both loci: rs288057 and rs288087 (both *P* = 0.002). The association remained statistically significant after Bonferroni correction (k=8, corrected *P* < 0.006). Notably, the A allele of rs288057 (OR = 1.43, 95%CI: 1.13-1.80) and the G allele of rs288087 (OR = 1.43, 95% CI: 1.13-1.80) were identified as risk alleles for SCZ (both *P* = 0.003).

**Table 3:** Distribution of genotypes and allele frequencies of the 8 SNPs of the *MAP2* gene in the control group and the SCZ group [n (%)].

SNPs	Genotype/Alle	HC	SCZ	OR (95%CI)	*χ^2^*	*P*	Padj 1
rs13017199	CC	282(69.1)	262(65.5)	1	2.721	0.257	0.129
	CG	117(28.7)	122(30.5)	1.122(0.828-1.522)			
	GG	9(2.2)	16(4.0)	1.913(0.831-4.405)			
	C	681 (83.5)	646 (80.8)	1	2.014	0.156	
	G	135 (16.5)	154 (19.3)	1.203 (0.932-1.552)			
rs561828230	CC	282(67.8)	295(71.3)	1	1.607	0.448	0.254
	CT	122(29.3)	111(26.8)	0.870(0.641-1.179)			
	TT	12(2.9)	8(1.9)	0.637(0.257-1.582)			
	C	686 (82.5)	701 (84.7)	1	1.475	0.225	
	T	146 (17.5)	127 (15.3)	0.851 (0.656-1.104)			
rs997109	TT	65(15.7)	43(10.4)	1	5.752	0.056	0.042^*^
	CT	196(47.5)	200(48.2)	1.542(1.001-2.378)			
	CC	152(36.8)	172(41.4)	1.711(1.099-2.663)			
	T	326 (39.5)	286 (34.5)	1	4.459	0.035^*^	
	C	500 (60.5)	544 (65.5)	1.240 (1.015-1.515)			
rs16843019	TT	19(4.6)	8(1.9)	1	5.949	0.051	0.042^*^
	CT	142(34.7)	134(32.2)	2.241(0.949-5.292)			
	CC	248(60.6)	274(65.9)	2.624(1.129-6.101)			
	T	180 (22.0)	150 (18.0)	1	4.075	0.044^*^	
	C	638 (78.0)	682 (82.0)	1.283 (1.007-1.634)			
rs2365661	AA	329(79.7)	325(77.8)	1	4.473	0.107	0.237
	AG	82(19.9)	84(20.1)	1.037(0.738-1.458)			
	GG	2(0.5)	9(2.2)	4.555(0.977-21.245)			
	A	740 (89.6)	734 (87.8)	1	1.326	0.250	
	G	86 (10.4)	102 (12.2)	1.196 (0.882-1.621)			
rs288057	GG	24(5.9)	12(2.9)	1	9.607	0.008^**^	0.002^**^
	AG	161(39.4)	138(33.0)	1.714(0.827-3.555)			
	AA	224(54.8)	268(64.1)	2.393(1.170-4.893)			
	G	209 (25.6)	162 (19.4)	1	9.052	0.003^**^	
	A	609 (74.4)	674 (80.6)	1.428 (1.132-1.802)			
rs288087	AA	25(6.0)	13(3.1)	1	9.284	0.010^*^	0.002^**^
	GA	160(38.6)	134(32.1)	1.611(0.793-3.271)			
	GG	230(55.4)	270(64.7)	2.258(1.129-4.514)			
	A	210 (25.3)	160 (19.2)	1	9.001	0.003^**^	
	G	620 (74.7)	674 (80.8)	1.427 (1.130-1.801)			
rs741006	AA	10(2.6)	3(0.8)	1	5.364	0.068	0.031^*^
	GA	116(29.7)	104(26.4)	2.989(0.801-11.155)			
	GG	264(67.7)	287(72.8)	3.624(0.987-13.310)			
	A	136 (17.4)	110 (14.0)	1	3.582	0.058	
	G	644 (82.6)	678 (86.0)	1.302 (0.990-1.711)			

^*^ *P*<0.05.

^**^ *P*<0.01.

*P*adj ^1^: adjusted for sex, age, smoking, alcohol consumption, and handedness (right-handed vs. non-right-handed). SCZ, schizophrenia; HC, healthy control.

Crucially, after adjusting for sex, age, smoking, alcohol use, and handedness, significant associations in genotype frequencies were observed for three additional loci: rs997109 (*P* = 0.042), rs16843019 (*P* = 0.042), and rs741006 (*P* = 0.031). Allele-based analysis confirmed the adjusted findings, identifying the C allele of rs997109 (OR = 1.240, 95%CI: 1.015-1.515, *P* = 0.035), the C allele of rs16843019 (OR = 1.283, 95%CI: 1.007-1.634, *P* = 0.044) as risk variants. However, after Bonferroni correction, neither the genotype nor the allele type of the above loci showed statistical significance between the case and control groups (*P*>0.006). No significant associations were observed for the remaining SNPs.

### 3.3 Association analysis between different genotypic models and SCZ

The results of analyses under different genotypic models are presented in **Table 4**. Significant differences between the case and control groups were observed for the recessive models of rs997109 (*P* = 0.022), rs16843019 (*P* = 0.028), rs2365661 (*P* = 0.035), and rs741006 (*P* = 0.048), as well as for both the dominant and recessive models of rs288057 (dominant: *P* = 0.006; recessive: *P* = 0.035) and rs288087 (dominant: *P* = 0.006; recessive: *P* = 0.045). No statistically significant differences were found for the remaining loci (all *P*>0.05). After adjusting for sex, age, smoking, alcohol consumption, and handedness, the dominant or recessive genotypic models of the above loci remained significantly different between the two groups: recessive models of rs997109 (*P* = 0.028), rs16843019 (*P* = 0.031), rs2365661 (*P* = 0.041), and rs741006 (*P* = 0.027); dominant and recessive models of rs288057 (dominant: *P* = 0.006; recessive: *P* = 0.033) and rs288087 (dominant: *P* = 0.005; recessive: *P* = 0.046). After Bonferroni correction, only the dominant models of rs288057 and rs288087 remained statistically significant (corrected *P* < 0.006).

**Table 4:** Genotypic model analysis of 8 SNPs in the *MAP2* between the control and SCZ groups.

SNP	A1	A2	TEST	SCZ (Comparison /Reference)	HC (Comparison/Reference)	*χ^2^*	*P*	Padj 1
rs13017199	G	C	DOM	138/262	126/282	1.202	0.273	0.229
rs13017199	G	C	REC	16/384	9/399	2.168	0.141	0.143
rs561828230	T	C	DOM	119/295	134/282	1.177	0.278	0.315
rs561828230	T	C	REC	8/406	12/404	0.800	0.371	0.400
rs997109	T	C	DOM	243/172	261/152	1.873	0.171	0.201
rs997109	T	C	REC	43/372	65/348	5.277	0.022^*^	0.028^*^
rs16843019	T	C	DOM	142/274	161/248	2.427	0.119	0.129
rs16843019	T	C	REC	8/408	19/390	4.828	0.028^*^	0.031^*^
rs2365661	G	A	DOM	93/325	84/329	0.452	0.501	0.506
rs2365661	G	A	REC	9/409	2/411	4.429	0.035^*^	0.041^*^
rs288057	G	A	DOM	150/268	185/224	7.495	0.006^**^	0.006^**^
rs288057	G	A	REC	12/406	24/385	4.460	0.035^*^	0.033^*^
rs288087	A	G	DOM	147/270	185/230	7.545	0.006^**^	0.005^**^
rs288087	A	G	REC	13/404	25/390	4.032	0.045^*^	0.046^*^
rs741006	A	G	DOM	107/287	126/264	2.489	0.115	0.090
rs741006	A	G	REC	3/391	10/380	3.906	0.048^*^	0.027^*^

^*^ *P*<0.05.

^**^ *P*<0.01.

*P*adj ^1^: adjusted for sex, age, smoking, alcohol consumption, and handedness (right-handed vs. non-right-handed). SCZ, schizophrenia; HC, healthy control; REC, recessive models; DOM, dominant models.

### 3.4 Linkage disequilibrium analysis of SNPs in the *MAP2*

**Figure 1:**
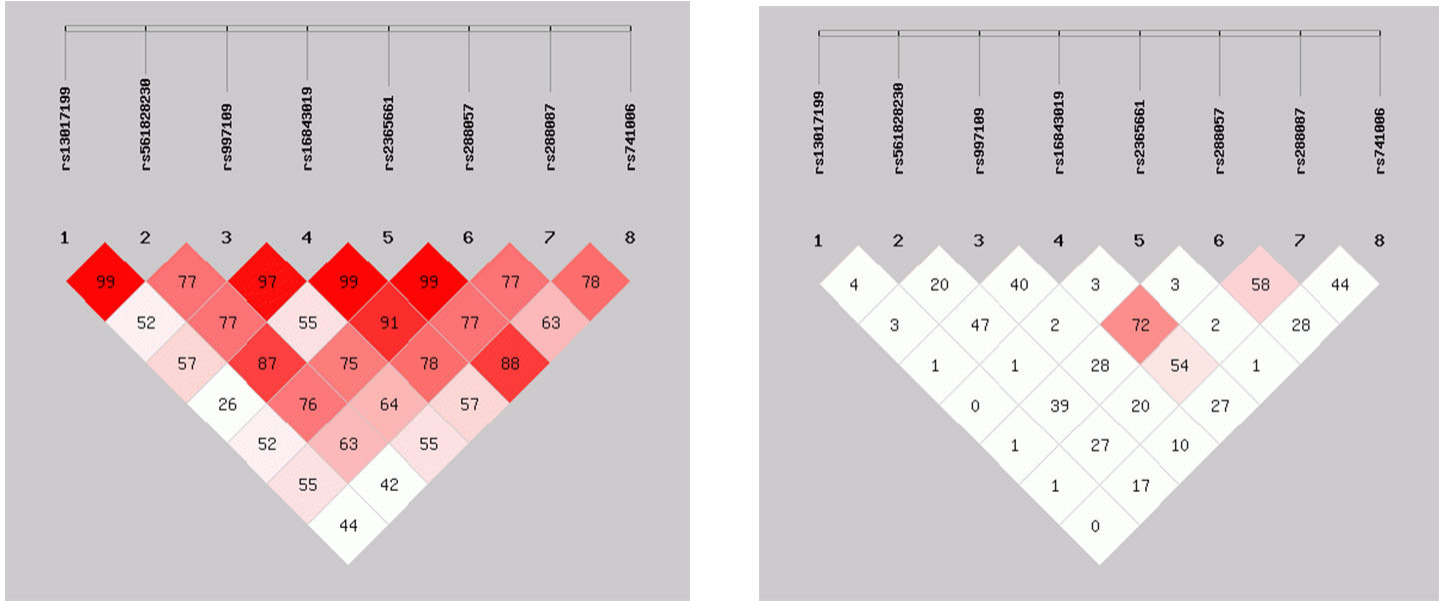
LD test for the 8 SNPs of *MAP2*. The plot was generated using the SHEsis platform. Left panel shows D’ values, and right panel shows r^2^ values. Values in the heatmap are presented multiplied by 100 (i.e., 28 = 0.28, 100 = 1.00). Color intensity provides a visual indication of the strength of LD: darker colors indicate stronger LD between two loci, whereas lighter colors reflect weaker LD (i.e., lower values).

The eight *MAP2* SNPs revealed strong LD between specific loci, most notably between rs16843019 and rs288057 (D′=0.919, r^2^=0.727) and between rs16843019 and rs288087 (D′=0.789, r^2^=0.543) (**Table 5**; **Figure 1**). These tightly linked SNPs were subsequently used to construct haplotypes for association testing.

**Table 5:** LD test for the 8 SNPs in the *MAP2*.

SNP	rs13017199	rs561828230	rs997109	rs16843019	rs2365661	rs288057	rs288087	rs741006
rs13017199	–	0.998	0.53	0.571	0.263	0.52	0.558	0.445
rs561828230	0.042	–	0.778	0.776	0.871	0.762	0.633	0.423
rs997109	0.036	0.203	–	0.97	0.551	0.757	0.643	0.558
rs16843019	0.018	0.473	0.4	–	0.998	0.919	0.789	0.578
rs2365661	0.002	0.019	0.023	0.032	–	0.998	0.772	0.882
rs288057	0.017	0.392	0.283	0.727	0.037	–	0.772	0.638
rs288087	0.019	0.274	0.202	0.543	0.022	0.587	–	0.788
rs741006	0.008	0.175	0.102	0.27	0.019	0.283	0.446	–

The values above the "-" represent D', and the values below the "-" represent r^2^.

Haplotype-based analysis identified significant case-control associations for specific haplotype blocks (**Table 6**). For the two SNP block rs16843019-rs288057, the frequencies of the CA (risk: OR = 1.432, 95% CI: 1.134-1.808, *P* = 0.003), CG (protective: OR = 0.499, 95%CI: 0.293-0.851, *P* = 0.009), and TG (protective: OR = 0.779, 95%CI: 0.608-0.999, *P* = 0.049) haplotypes differed significantly. For the rs16843019-rs288087 block, the CG haplotype constituted a risk factor (OR = 1.389, 95%CI: 1.111–1.735, *P* = 0.004), while the CA haplotype was protective (OR = 0.633, 95%CI: 0.411-0.974, *P* = 0.036). Furthermore, analysis of the three SNP block rs16843019-rs288057-rs288087 showed that the CAG haplotype was associated with increased risk (OR = 1.370, 95% CI: 1.071-1.753, *P* = 0.012), whereas the TGA haplotype was protective (OR = 0.753, 95%CI: 0.578-0.982, *P* = 0.036).

**Table 6:** Haplotype analysis of the *MAP2*.

SNP	Haplotype	HC (freq)	SCZ (freq)	χ2	P	OR (95%CI)
rs16843019-rs288057	C A	660.74(0.794)	595.41(0.730)	9.147	0.003^**^	1.432 (1.134-1.808)
	C G	21.26(0.026)	40.59(0.050)	6.743	0.009^**^	0.499 (0.293-0.851)
	T G	140.74(0.169)	168.41(0.206)	3.876	0.049^*^	0.779 (0.608-0.999)
rs16843019-rs288087	C A	36.24(0.044)	54.88(0.067)	4.381	0.036^*^	0.633 (0.411-0.974)
	C G	643.76(0.776)	582.12(0.713)	8.384	0.004^**^	1.389 (1.111-1.735)
	T A	123.76(0.149)	151.12(0.185)	3.851	0.050	0.771 (0.594-1.000)
	T G	26.24(0.032)	27.88(0.034)	0.085	0.771	0.923 (0.537-1.587)
rs16843019-rs288057-rs288087	C A A	25.17(0.030)	32.67(0.040)	1.465	0.226	0.722 (0.425-1.226)
	C A G	633.56(0.763)	561.75(0.690)	6.324	0.012^*^	1.370 (1.071-1.753)
	T G A	121.77(0.147)	145.88(0.179)	4.412	0.036^*^	0.753 (0.578-0.982)

^*^ *P*<0.05.

^**^ *P*<0.01.

SCZ, schizophrenia; HC, healthy control.

### 3.5 Genotype specific expression analysis

**Figure 2:**
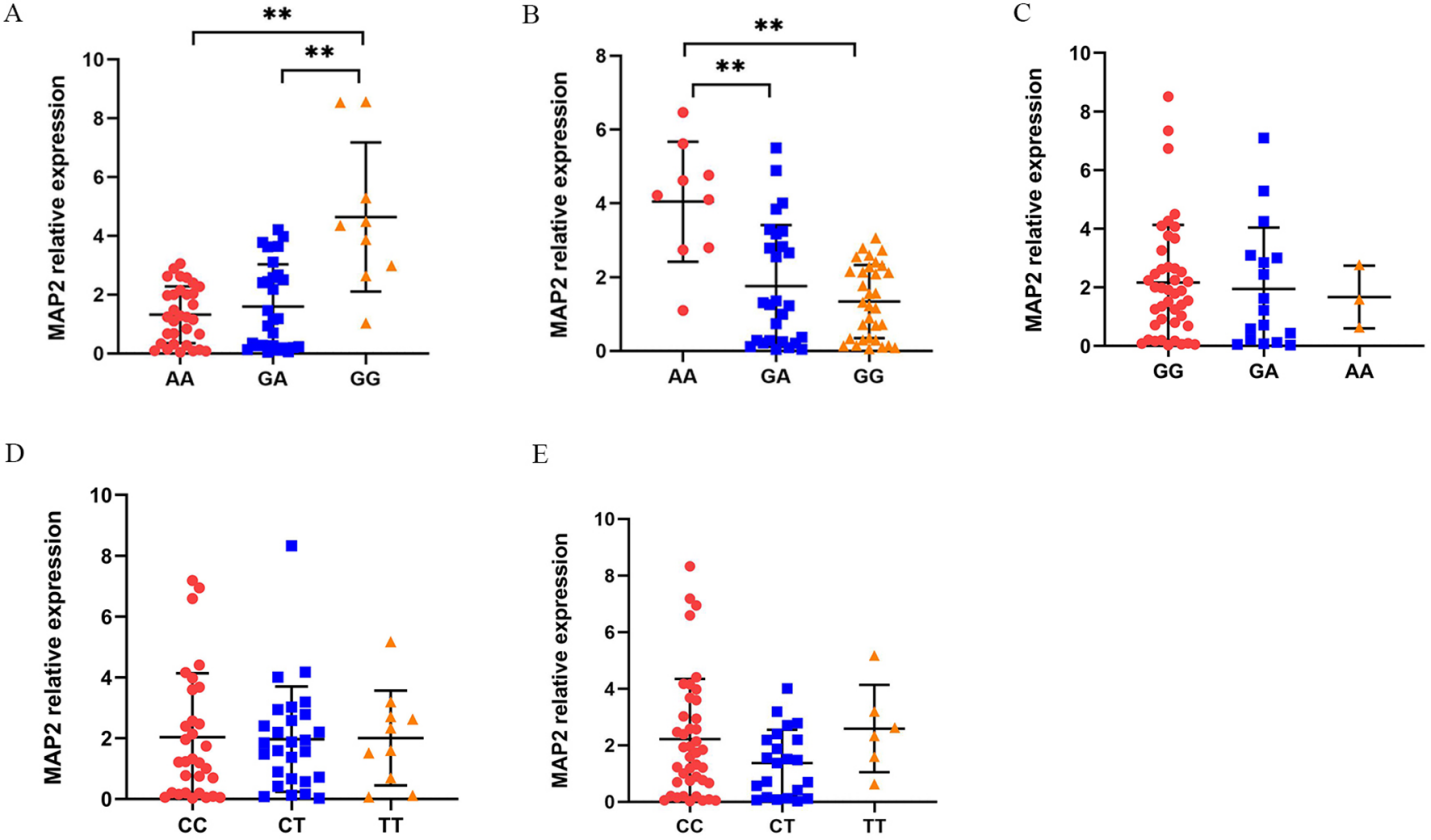
The expression levels of *MAP2* in SCZ patients with five SNP loci (^**^P < 0.01). **(A)** rs288057. **(B)** rs288087. **(C)** rs741006. **(D)** rs997109. **(E)** rs16843019.

To assess genotype specific effects on *MAP2* expression, we measured mRNA levels across genotypes for the five risk-associated SNPs in 69 patients with SCZ. Significant expression differences were observed at two loci: carriers of the AA genotype at rs288057 (AA vs GG: *P* = 5.45e-8; GA vs GG: *P* = 5.89e-7) (**Figure 2A**) and GG genotype at rs288087 (AA vs GA: *P* = 0.001; AA vs GG: *P* = 1.20e-5) (**Figure 2B**) showed significantly lower *MAP2* mRNA levels compared to other gennotypes carriers. In contrast, the other three SNPs, rs997109 (*P* = 0.992), rs16843019 (*P* = 0.161), and rs2365661 (*P* = 0.917), despite showing significant case-control frequency differences, had no significant effect on *MAP2* mRNA levels (**Figures 2C–E**).

### 3.6 *MAP2* expression profile and its link to clinical symptoms in SCZ

**Figure 3:**
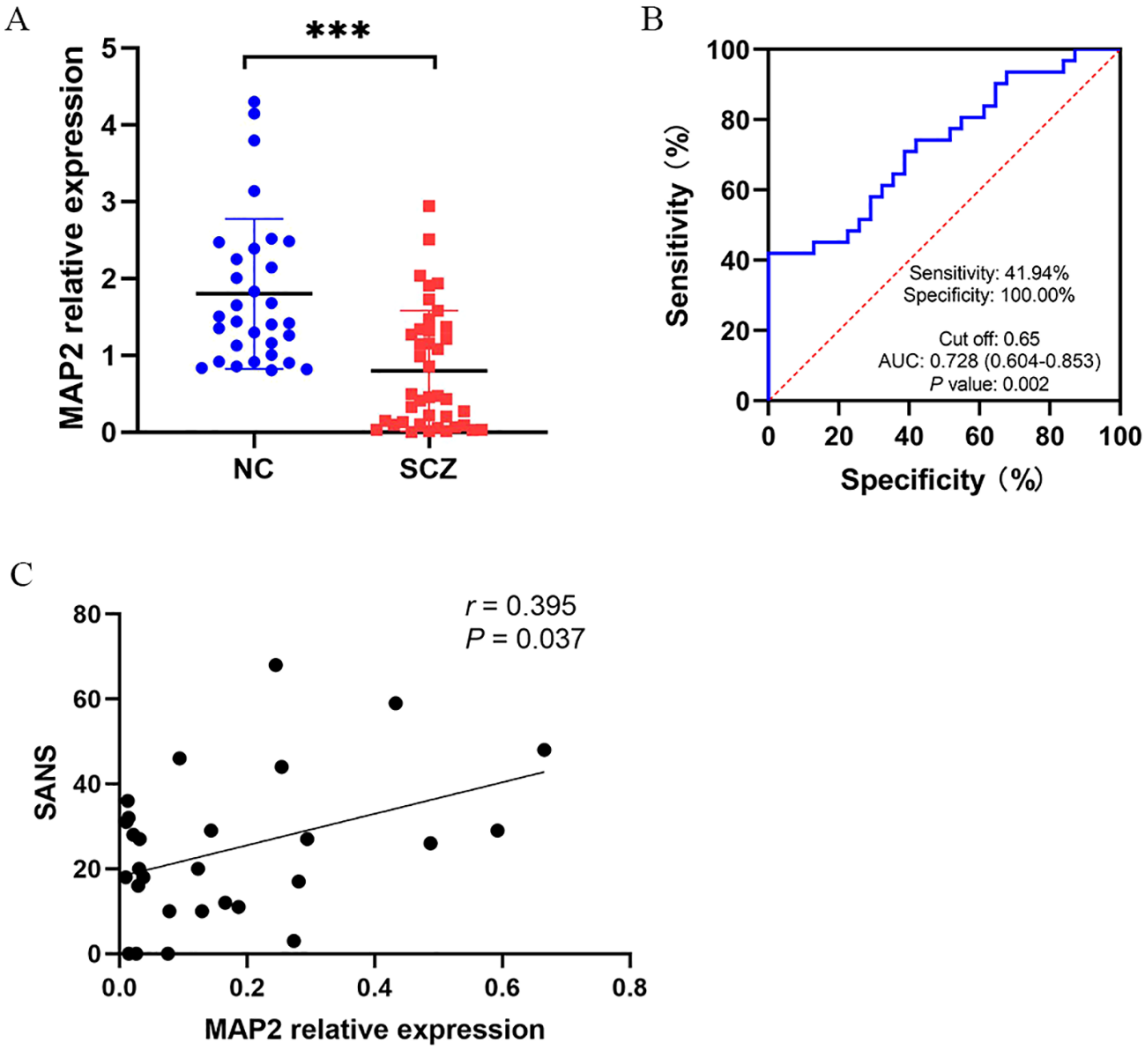
Analysis of *MAP2* gene mRNA expression and its correlation with clinical symptoms. **(A)** Expression levels of *MAP2* in healthy controls and schizophrenia patient (n=31:31;^***^P < 0.001). **(B)** Diagnostic value of *MAP2* gene expression level in SCZ. **(C)** Correlation between mRNA expression level of the *MAP2* gene and clinical symptoms (n=29).

To profile *MAP2* expression in SCZ, its mRNA levels were compared between 31 patients and 31 matched healthy controls. A significant downregulation was observed in the patient group (*P* = 0.001; **Figure 3A**). The diagnostic utility of *MAP2* expression was evaluated by ROC analysis, yielding an AUC of 0.728 (95% CI: 0.604-0.853; *P* = 0.002), with a specificity of 100% and a sensitivity of 41.9% (**Figure 3B**). Pearson correlation analysis further revealed a significant positive association between *MAP2* mRNA levels and the severity of negative symptoms assessed by the SANS (r=0.395, *P* = 0.037; **Figure 3C**; **Table 7**).

**Table 7:** Correlation between relative expression of *MAP2* and clinical scales in patients with schizophrenia.

Clinical Scale	M ± SD^1^	Pearson correlation		Multiple linear regression	
		*r*	*P*	*β*	*P*
BPRS	39.91±12.83	0.124	0.583	0.145	0.528
SAPS	17.07±13.81	0.191	0.329	0.239	0.213
SANS	24.46±17.27	0.395	0.037^*^	0.420	0.030^*^
YMRS	10.65±6.20	0.085	0.679	0.044	0.839
HAMD	13.88±7.15	0.205	0.316	0.374	0.097
HAMA	11.24±5.73	0.113	0.526	0.082	0.676

^1^ Data are presented as mean ± standard deviation (M ± SD).

^*^ *P* < 0.05.

To assess the independent association between *MAP2* expression and negative symptom severity while controlling for age, multiple linear regression analysis was performed with SANS score as the dependent variable, *MAP2* expression as the independent variable, and age as a covariate. Of note, all 29 participants included in this analysis were non-smokers, non-drinkers, and right-handed; thus, only age was entered as a covariate in the multiple linear regression model. The analysis revealed that *MAP2* expression remained significantly associated with SANS scores after adjusting for age (*β* = 0.420, *P* = 0.030).

### 3.7 Bioinformatic analysis of risk-associated SNPs

#### 3.7.1 Assessment of *MAP2* expression using public transcriptomic databases (GTEx and BrainSeq)

**Figure 4:**
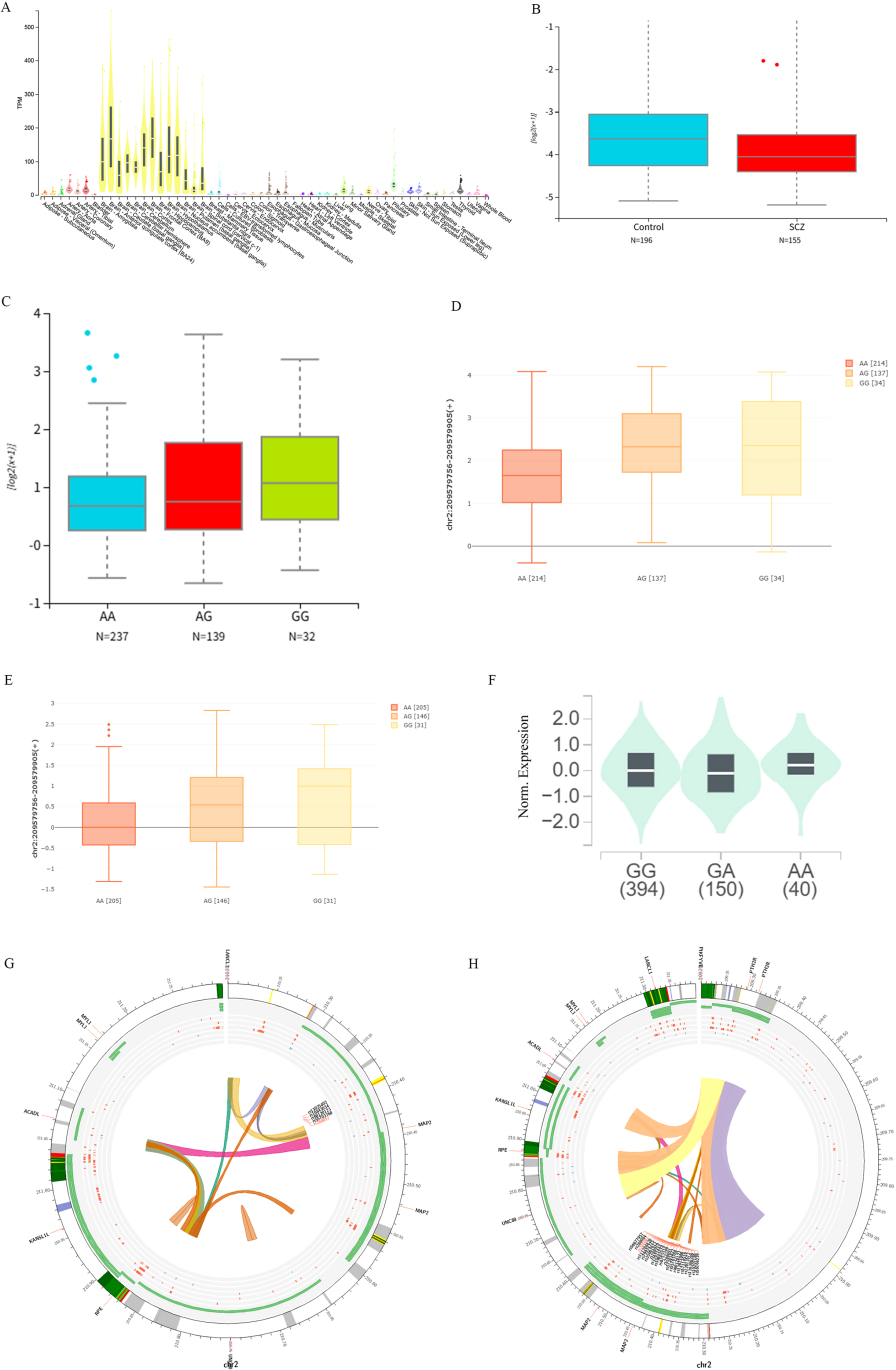
Genetic regulation and dysregulation of MAP2 in schizophrenia: from eQTLs to disease-associated expression. **(A)** Expression profile of the MAP2 gene in human tissues. **(B)** MAP2 expression is downregulated in the DLPFC of schizophrenia patients. **(C)** Correlation between rs288057 and MAP2 expression levels in the DLPFC (BrainSeq Phase 1). **(D)** Correlation between rs288057 and MAP2 expression levels in the DLPFC (BrainSeq Phase 2). **(E)** Correlation between rs288057 and MAP2 expression levels in the hippocampus (BrainSeq Phase 2). **(F)** Box plot of association analysis between genotypes at the rs288087 and MAP2 gene expression levels in visceral adipose tissue. **(G–H)** Circos diagram illustrating the genomic landscape of rs288057 **(G)** and rs288087 **(H)**. The circular and linear plots of chromatin loops, states and signatures associated to the SNP from outside to inside, they represent chromatin, annotation genes, histone (red), transcription factors (blue), current SNP and related SNP, and 3D chromatin interactions, respectively).

Interrogation of the GTEx database revealed ubiquitous *MAP2* expression across human tissues, with the highest levels observed in the brain (**Figure 4A**). Precomputed differential expression results retrieved from the dorsolateral prefrontal cortex (DLPFC) transcriptomic data from the BrainSeq database showed significantly lower *MAP2* expression in SCZ patients compared to healthy controls (log2FC =-0.345, *P* = 8.03e-4; **Figure 4B**).

#### 3.7.2 eQTL and chromatin interactions of rs288087 and rs288057 with *MAP2* from public databases

To determine if the SCZ risk-associated SNPs rs288087 and rs288057 correlated with *MAP2* expression, we extracted eQTL data for these variants from the public GTEx database. From BrainSeq RNA-seq data, a significant association was observed for rs288057 with *MAP2* expression in the DLPFC (Phase 1: Beta=0.390, *P* = 1.17e-6; Phase 2: Beta=0.230, *P* = 3.42e-9) and hippocampus (Phase 2: Beta=0.389, *P* = 4.42e-6), where the GG genotype was associated with higher expression than the AA genotype (**Figures 4C–E**). For rs288087, eQTL data retrieved from the GTEx database showed that its genotypes were significantly associated with *MAP2* expression in specific peripheral tissues; for example, the GG genotype was linked to lower expression in visceral adipose tissue compared to the AA genotype (*P* = 8.70e-5; **Figure 4F**). Based on chromatin conformation capture data, we identified distal genes (3D interacting genes) and SNP loci (3D interacting SNPs) that spatially interact with rs288087 and rs288057. Significant chromatin looping interactions were observed between these two SNPs and the *MAP2* gene across multiple neural and peripheral tissues, including the dorsolateral prefrontal cortex (Cortex_DLPFC), hippocampus, H1-NPC cells, and adrenal gland, with loop spans ranging from 100 to 200 kb. In addition, rs288087 and rs288057 were found to engage in chromatin loop interactions with several SNPs in linkage disequilibrium within neural tissues such as the hippocampus and adrenal gland (**Figures 4G, H**, the raw data are provided in **Supplementary Excel: Supplementary Tables S1-S4**).

#### 3.7.3 Regulatory element and transcription factor binding site annotation of rs288087 and rs288057

Functional annotation via HaploReg v4.1 positioned both SNPs within putative regulatory elements. The region encompassing rs288087 (risk allele: G) is predicted to possess enhancer activity and contain binding sites for regulatory proteins including CTCF (**Table 7**). Further interrogation of RegulomeDB ChIP-seq data confirmed that rs288087 resides within multiple CTCF binding peaks across various cell types (The raw data are provided in **Supplementary Excel: Supplementary Table 5**). Notably, in the brain-derived neuroblastoma cell line SK-N-SH, the CTCF binding signal intensity at this locus was high (594.59). Analysis of ENCODE chromatin state data revealed that the genomic region surrounding rs288087 exhibits a “Strong transcription” active state specifically in neural stem cells and neurons, in contrast to a “Quiescent/Low” state in most somatic cells (The raw data are provided in **Supplementary Excel: Supplementary Table 6**).

For rs288057 (risk allele: A), HaploReg and SNP2TFBS annotation indicated its location within an active enhancer region in blood cells, with predicted effects on the binding of transcription factors such as *HNF4*, *SOX10* and *Pou2f2* (**Table 8**). Consistent with this, motif analysis using RegulomeDB predicted that the allele variation at rs288057 could alter the binding affinity for the transcription factor *POU2F1* (*OCT1*).

**Table 8:** HaploReg v4.2 functional annotation results for SNP rs288087 and rs288057.

SNP	Risk allele	Protective allele	Enhancer histone marks	Proteins bound	Motifs changed
rs288087	G	A		CTCF	CTCF, HNF1, Hmbox1
rs288057	A	G	BLD		HNF4, Pou2f2,SOX10

## 4 Discussion

The current study conducts a comprehensive and multifaceted analysis, identifying the *MAP2* gene as a novel susceptibility locus for SCZ in the Chinese Han population. By integrating targeted genetic association analysis with functional investigation of gene expression, we have proposed a coherent pathological pathway linking specific genetic variations to a potentially plausible molecular mechanism underlying the disorder.

Our targeted genetic analysis identified multiple SNPs within the *MAP2* significantly associated with SCZ risk. Although *MAP2* has not been highlighted as a genome-wide significant locus in the largest SCZ GWAS to date, this may be due to modest common variant effects, population heterogeneity, or epigenetic modification, that are not captured by GWAS. Our finding is consistent with the broader observation from large-scale genomic studies that synaptic and cytoskeletal pathways, rather than specific genes, are enriched among SCZ risk loci (3–5). This apparent discrepancy may be explained, at least in part, by the fact that *MAP2* is an intrinsically disordered protein whose function is heavily regulated by post-translational modifications (e.g., phosphorylation) and epigenetic mechanisms, which are invisible to conventional GWAS approaches (8, 9). The convergence of association signals across several SNPs within the same gene strengthens the evidence against a spurious finding and underscores the potential etiological importance of this genomic region. Notably, rs288057 and rs288087 emerged as prime functional candidates for subsequent investigation. Haplotype analysis further refined this genetic architecture, revealing distinct risk and protective haplotype blocks. For instance, the CG haplotype (rs16843019-rs288057) exhibited a significant protective effect. This observation suggests disease risk is modulated by specific allelic combinations through complex cis-interactions that fine-tune gene regulation, rather than by isolated variants alone (29). The apparent protective effect of a haplotype containing an individual risk allele highlights the non-additive and compensatory interactions possible within regulatory haplotypes.

A pivotal advance of this study is the functional convergence of schizophrenia-associated genetic variants on the dysregulation of *MAP2*. We established that risk variants at rs288057 and rs288087, function as cis-regulatory elements influencing *MAP2* transcription in disease-relevant contexts. This is supported by consistent genotype-expression correlations for rs288057 in SCZ-affected brain regions and by the co-localization of both SNPs within the three-dimensional chromatin interactome of the *MAP2* promoter. These findings set the stage for elucidating distinct, synergistic mechanisms underlying *MAP2* dysregulation in SCZ.

To delineate the distinct molecular pathways, our multi-source bioinformatic analyses revealed that rs288057 and rs288087 converge on *MAP2* downregulation through fundamentally different mechanisms, together forming a coherent “dual-regulation” model relevant to neurodevelopment. For rs288057, located in a promoter-proximal region with a “strong transcription” chromatin state in neural stem cells, motif analyses (RegulomeDB, Haploreg, and SNP2TFBS) predict that its risk allele disrupts binding sites for neural transcription factors, including *POU2F2*, *SOX10* and *HNF4* (30–33). *SOX10* is crucial for glial differentiation and myelination, processes implicated in SCZ pathology (32). The developmental-stage-specific activity of this region suggests rs288057 may impair the initiation of *MAP2* transcription during critical neurodevelopmental windows. Critically, chromatin conformation capture (3C/Hi-C) data physically links both SNP loci to the *MAP2* promoter via chromatin loops in multiple neural tissues, including the DLPFC and hippocampus. This direct spatial evidence confirms their roles as integral components of the *MAP2* regulatory landscape. Within this architecture, rs288087 resides in a neural-specific enhancer loop anchor, embedded within a high-confidence CTCF binding peak (34, 35). We thus hypothesize that the risk allele may disrupt this CTCF-mediated looping, decoupling the enhancer from its target promoter. Intriguingly, the mechanistic dichotomy, where rs288057 affects transcription factor binding motifs while rs288087 impacts higher-order chromatin structure, is reflected in their respective bioinformatic predictions. Collectively, we propose that rs288057 and rs288087 jointly ensure precise spatiotemporal *MAP2* expression: the former potentially gates the “timing” (developmental activation) and the latter the “spatial” fidelity (chromatin looping) of transcription. This work transforms genetic associations into a physically grounded and testable, neurodevelopmentally framed hypothesis for synaptic dysfunction in SCZ, providing a crucial mechanistic framework for future functional studies (9).

The genetically driven reduction in *MAP2* expression provides a compelling upstream explanation for one of the most consistent neuropathological hallmarks of schizophrenia. Our data align with post-mortem literature documenting decreased *MAP2* protein and profound dendritic spine pathology in brain regions critical for cognition and emotion, such as the prefrontal cortex and hippocampus (7, 23–25). *MAP2* is a master regulator of dendritic homeostasis, stabilizing microtubules, facilitating synaptic component trafficking, and scaffolding signaling molecules (9, 36). Therefore, reduced *MAP2* abundance would be predicted to destabilize dendritic arbors and impair spine formation and maintenance. This cytoskeletal instability would in turn impair the proper delivery and anchoring of postsynaptic density proteins, thereby compromising spine formation and maintenance. Ultimately, these structural deficits are likely to dysregulate the precise, activity dependent synaptic signaling that underlies learning and circuit refinement. These cellular phenotypes are central to the circuit dysconnectivity hypothesis of SCZ and represent convergent pathological features across neuropsychiatric disorders (37, 38). Our findings thus posit that *MAP2* risk variants constitute a primary, predisposing insult that initiates or exacerbates this cascade of cytoskeletal and synaptic dysfunction.

The transcriptional dysregulation of *MAP2* proposed here may underpin its altered expression observed in patients, yet its relationship with clinical symptoms reveals additional complexity. While *MAP2* mRNA was significantly downregulated in schizophrenia patients overall, within the patient cohort, its expression level showed a positive correlation with the severity of negative symptoms. Although this appears counterintuitive, a remarkably similar pattern has been reported in postmortem brain studies of schizophrenia. Rosoklija et al. examined immunohistochemistry for *MAP2* in the hippocampal subiculum of 64 psychiatric patients and found that a prominently depressed expression level of *MAP2* in 20% of schizophrenia cases was associated with fewer positive and negative symptoms over the course of the illness (39). The authors proposed that this reduction in *MAP2* may represent an adaptive response to schizophrenia (39). We therefore propose a parallel interpretation: the overall *MAP2* deficit reflects disease-related pathology, whereas the positive correlation among patients may represent a maladaptive compensatory upregulation in those with more severe negative symptoms. In this view, greater symptom severity triggers an attempt to stabilize microtubule function and dendritic integrity, but this response is ultimately insufficient to restore normal neuronal function, a failure of homeostatic adaptation. Additionally, our measurement of *MAP2* in peripheral blood, rather than brain tissue, may capture indirect or systemic signals that do not linearly reflect central pathology. Future studies using brain tissue or neuron-derived exosomes are needed to validate these findings. Therefore, our findings delineate a pathway from genetic risk to gene dysregulation, and further link the magnitude of this dysregulation *in vivo* to the clinical severity of core, treatment-resistant features, highlighting *MAP2* not only as a disease vulnerability factor but also as a potential dynamic marker of pathological state.

Our findings have two primary translational implications. First, the downregulation of peripheral *MAP2* mRNA demonstrated potential as a biomarker with high specificity but moderate sensitivity. This profile suggests that while not a universal feature, low *MAP2* expression is strongly associated with the disease state in a subset of patients. It may therefore hold utility for biological subtyping identification within a stratified psychiatry framework (40). Second, the complex link between *MAP2* expression and negative symptoms may reflect a maladaptive molecular response to severe circuit dysfunction. This identifies cytoskeletal regulation and dendritic integrity as tangible targets for novel therapeutic development aimed at this debilitating symptom domain.

Positioning our work within the broader literature, large-scale GWAS have implicated the broader chromosomal region containing *MAP2*, but pinpointing causal genes and variants remains challenging. Our hypothesis driven candidate gene approach, motivated by strong prior neuropathological evidence, allowed us to focus on *MAP2* and provide direct evidence linking risk alleles to reduced expression and clinical symptoms. This integration of genetics with molecular and clinical phenotypes adds a crucial functional layer to the statistical signals from genome-wide studies, generating a specific and testable mechanistic hypothesis.

While this study provides convergent, multi-level evidence, several limitations must be acknowledged. First, our primary functional correlation relies on mRNA from peripheral blood leukocytes. Although this accessible source shows concordance with known brain pathology (41, 42), direct validation in disease-relevant neural tissues is essential to confirm causality and examine downstream cellular phenotypes. Second, and relatedly, the proposed regulatory mechanisms for rs288057 and rs288087, while strongly supported by bioinformatic and chromatin interaction data, require direct experimental validation to confirm their functional impact. Third, regarding population and power: we did not perform PCA to control for population stratification, all participants from same self-reported Han Chinese regional population, but residual stratification remains possible; sample sizes varied across analyses (e.g., n=31/31 for ROC analysis), limiting statistical power for higher-order interactions; and key clinical variables (e.g., illness duration, age at onset, treatment status, hospitalization) were not systematically reported, which may introduce confounding. Fourth, as a case-control study, we cannot establish the temporal sequence. Longitudinal studies in clinical high-risk individuals are needed to determine if *MAP2* downregulation precedes illness onset, thereby evaluating its potential as a predictive biomarker; and the claim of a diagnostic biomarker is premature given the modest AUC (0.728), low sensitivity (41.9%), small sample, and lack of external validation. Future studies with larger, independent, and diverse cohorts, incorporating ancestry markers and comprehensive clinical characterization, are needed to validate our findings.

In conclusion, this study identifies *MAP2* as a susceptibility gene for schizophrenia. We propose that non-coding risk variants such as rs288057 and rs288087 contribute to disease by reducing *MAP2* expression. This downregulation provides an upstream explanation for the dendritic and synaptic deficits characteristic of the disorder. Our findings thus bridge a genetic risk factor to a specific molecular pathway and core clinical symptoms, highlighting cytoskeletal regulation as a critical focus for understanding disease mechanisms and future therapeutic development.

## Data Availability

The raw data supporting the conclusions of this article will be made available by the authors, without undue reservation.
